# miR-27a-5p, miR-21-5p, miR-1246 and miR-4508: a candidate microRNA signature in the protection and regulation of viral infection in mild COVID-19

**DOI:** 10.1186/s10020-025-01154-0

**Published:** 2025-03-15

**Authors:** Malena Gajate-Arenas, Candela Sirvent-Blanco, Omar García-Pérez, Angélica Domínguez-de-Barros, José E. Piñero, Jacob Lorenzo-Morales, Elizabeth Córdoba-Lanús

**Affiliations:** 1 Instituto Universitario de Enfermedades Tropicales y Salud Pública de Canarias (IUETSPC), Universidad de La Laguna, La Laguna, Tenerife 38029 Spain; 2 Centro de Investigación Biomédica en Red de Enfermedades Infecciosas (CIBERINFEC), Instituto de Salud Carlos III, Madrid, 28029 Spain; 3 Departamento de Obstetricia y Ginecología, Pediatría, Medicina Preventiva y Salud Pública, Toxicología, Medicina Legal y Forense y Parasitología. Facultad de Ciencias de la Salud, Universidad de La Laguna, San Cristóbal de La Laguna, Tenerife 38200 Spain

## Abstract

MicroRNAs (miRNAs) are gene regulators essential for cell homeostasis, their alteration is related to a pathological state, including infectious diseases like COVID-19. Identifying an altered profile of circulating miRNAs in mild COVID-19 may enhance our knowledge of the pathogenesis of SARS-CoV-2 and the range of clinical phenotypes. In the present study, a miRNA screening was performed by Next Generation Sequencing (NGS), and the expression levels of 13 resulting miRNAs were validated through RT-qPCR in the serum of 40 mild cases compared to 29 non-infected individuals. An in-silico analysis was performed to detect target genes and their related pathways. From the validated miRNAs, miR-1246 (*p* < 0.001), miR-423-5p (*p* < 0.001), miR-21-5p (*p* = 0.005), miR-146a-5p (*p* < 0.001), miR-4508 (*p* = 0.001), miR-629-5p (*p* < 0.001), and miR-210-3p (*p* = 0.002) were found downregulated in infected individuals. Only miR-27a-5p was overexpressed in subjects with COVID-19 (*p* = 0.013) and associated with SARS-CoV-2 infection (*p* = 0.010). The KEGG pathways and GO analysis revealed that the differentially expressed miRNAs were related to viral processes or immunological pathways: miR-27a-5p acts on the TGF-beta pathway; miR-21-5p targets *SMAD7*, which is associated with the inflammatory response in the lung; miR-1246 acts on p53 pathway; and miR-4508 acts on *ICAM2*. In conclusion, the most relevant miRNAs, miR-27a-5p and miR-21-5p, were differently expressed in mild forms of COVID-19. The higher expression of miR-27a-5p observed in mild COVID-19 cases may suggest a protective effect against severe forms of the disease. Reduced expression of miR-21-5p may prevent pulmonary inflammation and the progression of fibrosis. The downregulation of miR-1246 and miR-4508 in mild COVID-19 cases may conduct the correct control of the infection. Moreover, miR-423-5p might be a suitable biomarker in the early stages of SARS-CoV-2 infection.

**Supplementary Information:**

The online version contains supplementary material available at 10.1186/s10020-025-01154-0.

## Introduction

SARS-CoV-2 is a virus that belongs to the Coronaviridae family and is the infectious agent that causes COVID-19. This family, well-known for causing respiratory infections in mammals and birds, is often involved in stationary mild colds. However, two coronaviruses were able to cause epidemic outbreaks: the severe acute respiratory syndrome (SARS) and the Middle East respiratory syndrome (MERS)(Ovsyannikova et al. 2020; V’kovski et al. 2021; Wiersinga et al. 2020). SARS-CoV-2 shows an efficient transmission, promoting the fast spread of the virus and causing a pandemic outbreak in 2020. The World Health Organization (WHO) declared it a global health emergency until May 2023 (Deng et al. 2021; Hu et al. 2021; Wiersinga et al. 2020).

The coronavirus disease COVID-19 presents a wide variety of symptoms, with the most common being dry cough, fever, and fatigue. While most cases present mild symptoms, the infection can progress to severe forms of the disease (Wiersinga et al. 2020). SARS-CoV-2 targets the epithelial cells from the respiratory tract, where it replicates, and then migrates to the lungs (Hu et al. 2021; V’kovski et al. 2021). If the immune response is not well-coordinated, an excessive influx of immune cells gets into the lungs and promotes tissue damage instead of relieving the infection. This heightened immune response is caused by an acute increase in pro-inflammatory cytokines, known as “Cytokine Storm”, which can lead to respiratory distress syndrome, followed by respiratory failure and multi-organ failure (Hu et al. 2021; Jiang et al. 2022; Ragab et al. 2020).

In this scenario, patients can vary from asymptomatic or mild cases to severe forms that may progress to require hospitalisation and Intensive Care. So is the importance of understanding why certain people are more predisposed to developing severe forms of the disease. Investigations have shown that people over the age of 60, especially men, are at a higher risk of developing severe COVID-19 (Grifoni et al. 2023; Tharakan et al. 2022). Additionally, patients with underlying diseases such as diabetes and hypertension are more likely to develop severe outcomes (Chenchula et al. 2023; Khan et al. 2020). In this context, certain clinical features, like lymphopenia, RNAaemia, leukocytosis, etc., can help predict severe outcomes (Gutiérrez-Pérez et al. 2022; Huang et al. 2020; Zinellu et al. 2021). However, these parameters may manifest in the blood test late and may not fully explain the biology of the virus and the infectious process.

In recent years, small RNAs have emerged as promising tools for advancing research and therapeutic applications. MicroRNAs (miRNAs) are 17 to 25 nucleotides long and single-stranded. They act as gene regulators by reducing or increasing the expression of target genes (Winkle et al. 2021). As prognostic biomarkers, they have shown their sensitivity in detecting early tumors and metastasis in cancer (Mohr and Mott 2015). In respiratory infections, miRNAs are essential in the host response to fight against the virus. However, viruses can alter miRNA expression levels, making the cells more suitable for viral replication (Tahamtan et al. 2016).

In COVID-19, some studies have found altered miRNA expression in serum samples from individuals infected by SARS-CoV-2 (Pollet et al. 2023). Moreover, the over-expression and/or downregulation of certain miRNAs have been associated with worse outcomes in COVID-19 (Giannella et al. 2022; Mohamed et al. 2023) However, there is scarce information regarding mild cases. Identifying an altered profile of circulating miRNAs in serum samples of individuals with mild COVID-19 might improve our understanding of the pathogenesis of SARS-CoV-2 and shed light on the diversity of phenotypes in clinical manifestations.

## Materials and methods

### Individuals included in the study

In this study, 40 mild symptomatic cases, with a molecular diagnosis of COVID-19 were included. These cases were recruited at the University of La Laguna, and private laboratories in Tenerife, Spain. Serum samples were collected at the time of diagnosis coinciding with the first seven days of infection or at the beginning of symptoms. The subjects under study were males and females with a wide range of ages. Twenty-nine healthy individuals with a negative diagnosis of COVID-19, matched in age and sex to the cases, were included as a control group. Subjects with underlying diseases or undergoing medical treatment were excluded from the study. All the individuals included in the study were fully vaccinated at the time of sample collection. The study was approved by the ethical committee board from Hospital Universitario de Canarias, and written informed consent was obtained from all participants (CHUC B1947). This study was conducted following the Declaration of Helsinki.

Serum samples were separated from whole blood within 1 h after collection and stored at − 80 °C until further use in the genetic study.

### miRNA screening by next generation sequencing

Serum samples corresponding to eight individuals (4 cases with COVID-19 and 4 controls) were analysed in the miRNA screening step by Next Generation Sequencing (NGS). RNA was isolated from serum samples using the miRNeasy Serum/Plasma Advanced Kit (Qiagen) and converted into miRNA NGS libraries using the QIAseq miRNA Library Kit (QIAGEN). Sample QC quality (QC) was checked using a qPCR assay to determine if miRNAs hsa-miR-103a-3p, hsa-miR-191-5p, hsa-miR-451a, hsa-miR-23a-3p and hsa-miR-30c-5p expression, were within the expected range. Samples were also checked for inhibition of enzymatic reactions (spike-in control UniSp6) and potential hemolysis (miR23a, miR451a).

The resulting library pools were sequenced on a NextSeq Illumina platform (Illumina, Inc.). The numbers of known miRNAs were calculated by counting data to relevant entries in miRBase v22 software (http://mirbase.org). The miRNA expression was expressed as Tags Per Million (TPM, the number of reads for a particular miRNA). The NormFinder software was used to identify miRNAs stably expressed across all samples for their use as candidate reference genes (http://moma.dk/normfinder-software).

### miRNA validation by qPCR

The top abundant miRNAs resulting from the NGS analysis were validated at this step, by RT-qPCR in the 40 COVID-19 cases and 29 non-COVID-19 controls. Moreover, miR-21-5p, miR-155-5p, miR-210-3p and miR-146a-5p were included due to their relevance in COVID-19 pathogenesis described in other studies (Martínez-Fleta et al. 2022; Sabbatinelli et al. 2021; Tang et al. 2020). The miRNAs extraction from serum was carried out using miRNeasy Serum/Plasma Advanced Kit (Qiagen, Germany), and its quality was evaluated by NanoDrop Lite (ThermoScientific, USA). The relative gene expression analysis was set up in a two-step RT-qPCR. First, miRNAs were retrotranscribed into cDNA using miRCURY LNA RT Kit (Qiagen, Germany), and its expression was determined by a qPCR by using the miRCURY LNA SYBR Green PCR Kit (Qiagen, Germany) and miRCURY LNA miRNA PCR Assays (Qiagen, Germany) (Supplementary Table 1). The reaction was performed in a real-time qPCR machine StepOne Plus (Applied Biosystem, ThermoFisher Scientific, Massachusetts, USA). Each reaction was performed in duplicate, setting up the experiment in 40 cycles. miR-26a-5p, the best candidate as a reference gene in the NGS screening analysis, was used for data normalisation. A non-template control was carried out in each experiment. The relative expression analysis of the target genes was determined using the comparative threshold method 2^ΔΔ^Ct.

### In-silico analysis

The resulting dysregulated miRNAs were integrated to search for potential targets in three software’s: miRBase v22 (http://www.mirbase.org/), TargetScan v.8.0 (http://www.targetscan.org/vert_80/), and the DIANA tool miRPath-v4.0 (https://dianalab.e-ce.uth.gr/html/mirpathv4/). The g: Profiler software (https://biit.cs.ut.ee/gprofiler/gost) was used to contrast the resulting target genes proposed.

Functional and enrichment analyses were performed by using Gene-ontology (GO) terms (Ashburner et al. 2000) and the Kyoto Encyclopaedia of Genes and Genomes (KEGG) (https://www.kegg.jp/).

### Statistical analysis

Continuous variables were described using means, standard deviations or medians and percentiles P_25_; P_75_ when not normally distributed. In the analysis of the miRNA sequencing, the Empirical analysis of DGE algorithm Bioconductor software was used for differential expression analysis with default settings (Robinson et al. 2010). The R package DESeq2 version 1.28.1 was used (Love et al. 2014; Team R Core and others 2013). The Benjamini–Hochberg false discovery rate (FDR) algorithm was used for multiple testing corrections.

The distribution of gene expression was analyzed using the Kolmogorov-Smirnov test. Data were normalized using the method of the log two-fold and absolute gene-wise changes in expression (Log2FC). A miRNA was considered a candidate for validation when its Log2FC > 1.5. The Mann- Whitney U test or the t-test t was used for group comparisons as appropriate. Chi-squared test was performed to determine significant differences between cases and controls concerning sex. The correlation between age and miRNA expression was analysed by Spearman’s rank correlation coefficient or Pearson’s rank correlation coefficient as appropriate.

To determine predictor variables for cases and controls a binary logistic regression model, one for each, was fitted. Age, sex, and normalized gene expression were considered as predictor variables. Odds ratios (OR) with a 95% confidence interval were reported. The Hosmer and Lemeshow goodness-of-fit test (*p* > 0.05) was used to check model fitness. For all analyses, significance was set at *p* < 0.05. The statistical analysis was performed using SPSS v. 25 (IBM Corp, USA) and GraphPad Prism v. 9.4.1 (Dotmatics, UK) software.

## Results

### miRNAs screening results

Eight individuals, comprising four mild COVID-19 cases and four age-matched controls without a SARS-CoV-2 infection were screened for miRNA expression. All cases and controls were male and age-matched (mean age of 56 years).

**Fig. 1:**
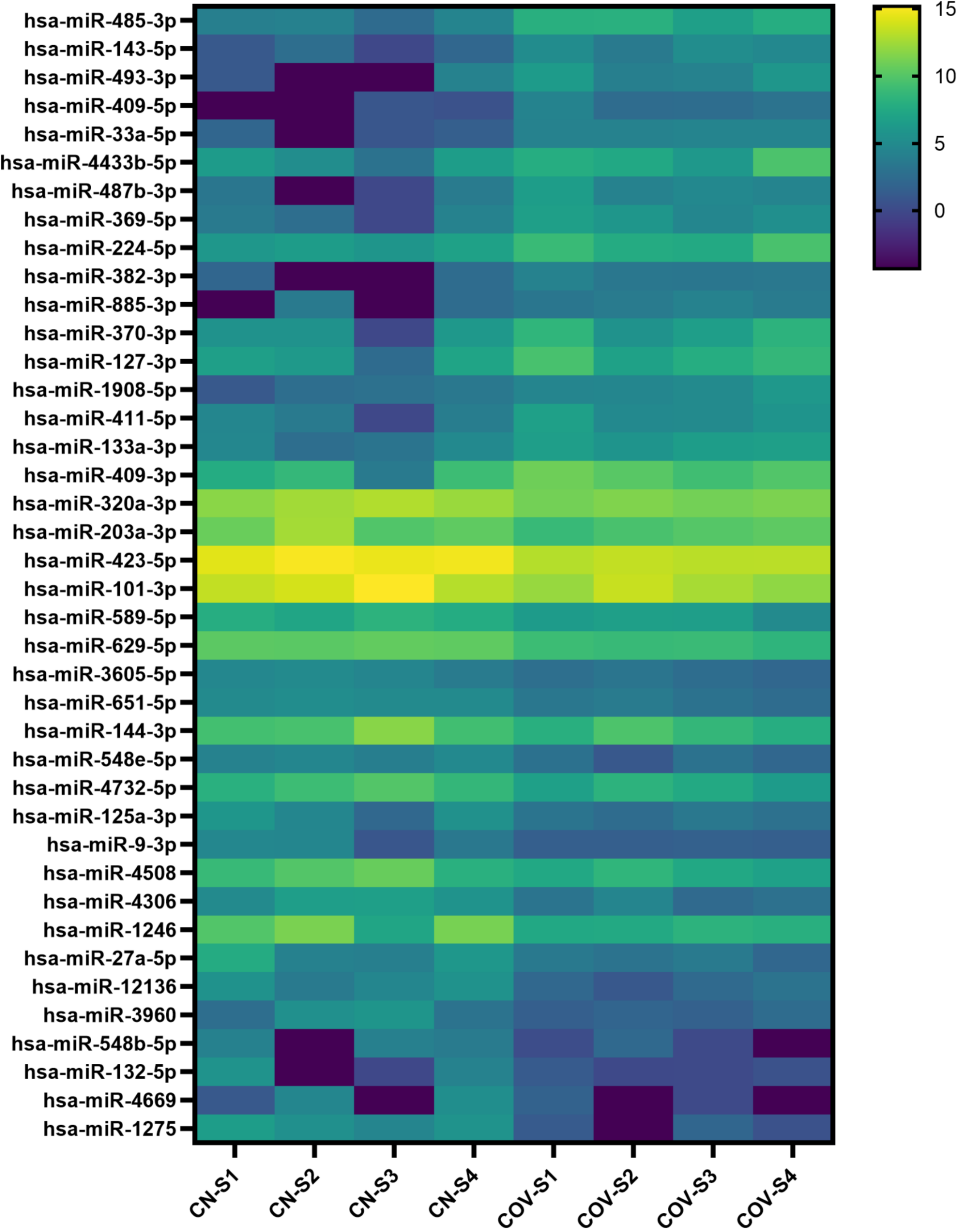
Heatmap showing the top 40 significantly dysregulated miRNAs between COVID-19 cases and controls (FDR < 0.05). Each row represents one miRNA, and each column represents one sample (cases: COV-S1 to COV-S4 and controls: CN-S1 to CN-S4). Yellow indicates upregulated genes and dark blue downregulated genes

**Fig. 2:**
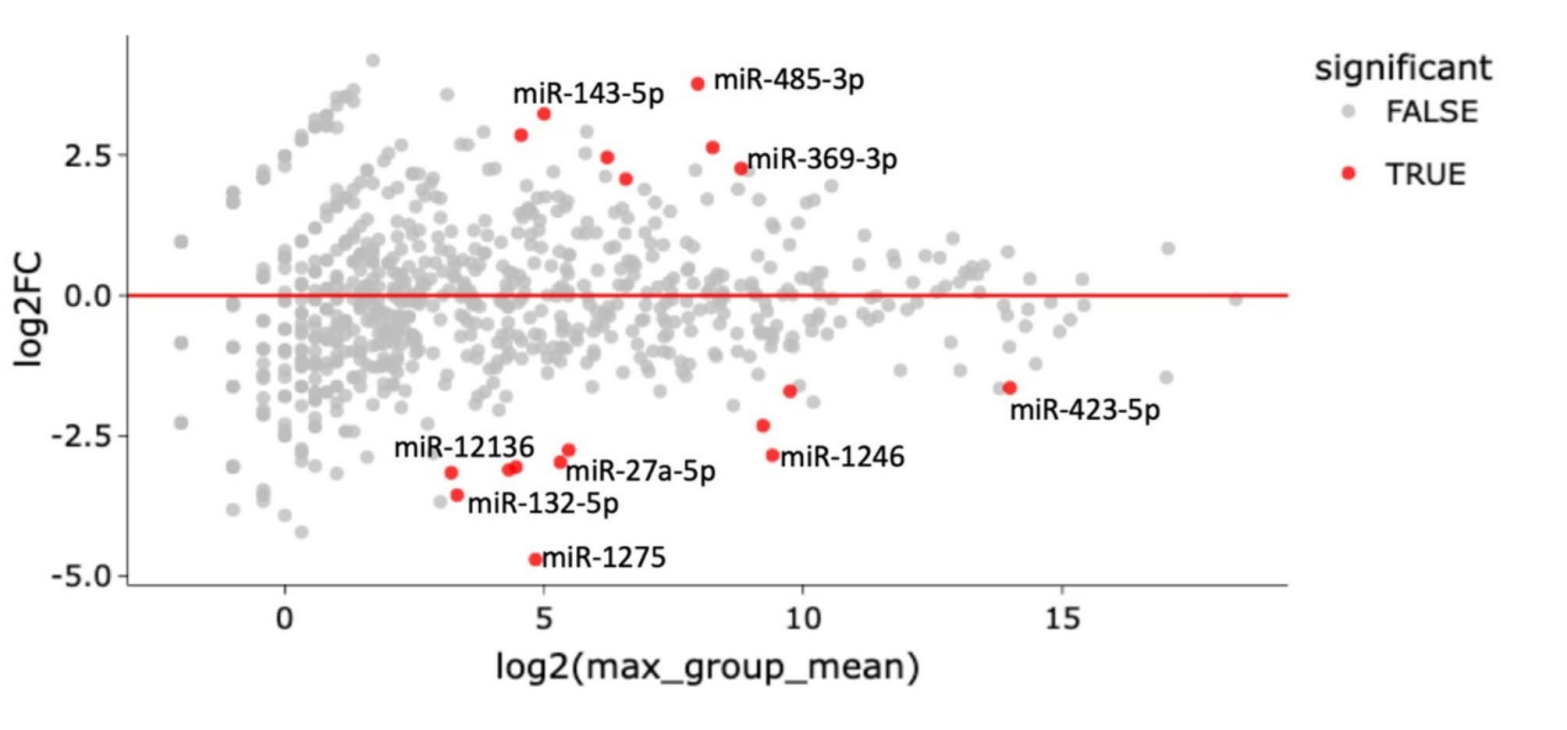
Each gene’s fold change (FC) is plotted against its mean expression among all samples. All significantly differentially expressed genes are marked in red. Significant changes are defined as *p*-value < 0.001, FDR < 0.01, and Log2FC > 2

Over one hundred and seventy miRNAs (≥ 10 TPM) were identified by next-generation sequencing (NGS) in the analysed samples. The top 40 dysregulated miRNAs are represented in Fig. 1. Eighteen miRNAs were found to be significantly dysregulated in the serum from mild symptomatic COVID-19 cases when compared to healthy controls (*p*-value < 0.001; FDR < 0.01) (Fig. 2 and Table 1). miR-485-3p and miR-143 were the most up-regulated (Log2FC = 3.77 and 3.23, FDR = 2.2e-7 and 0.0018, respectively) ones while miR-132-5p and miR-1275 were the most down-regulated (Log2FC=-3.55 and − 4.70, FDR = 0.0031 and 5.2e-8, respectively).

**Table 1:** Main dysregulated circulating miRNAs in the study population resulting from the NGS screening

Name	Log2FC	*p*-value	FDR
hsa-miR-485-3p	3.77	8.2e-10	2.2e-7
hsa-miR-143-5p	3.23	0.000021	0.0018
hsa-miR-33a-5p	2.85	0.00018	0.0062
hsa-miR-4433b-5p	2.63	0.00019	0.0062
hsa-miR-369-5p	2.46	0.00027	0.0079
hsa-miR-224-5p	2.26	0.000079	0.0035
hsa-miR-133a-3p	2.07	0.00012	0.0045
hsa-miR-423-5p	-1.64	0.000064	0.0031
hsa-miR-629-5p	-1.70	0.00027	0.0079
hsa-miR-4508	-2.31	0.000095	0.0038
hsa-miR-4306	-2.75	0.000027	0.0020
hsa-miR-1246	-2.84	0.0000011	0.00014
hsa-miR-27a-5p	-2.97	0.000012	0.0012
hsa-miR-12,136	-3.05	4.2e-7	0.000073
hsa-miR-3960	-3.10	0.000044	0.0025
hsa-miR-548b-5p	-3.15	0.000042	0.0025
hsa-miR-132-5p	-3.55	0.000065	0.0031
hsa-miR-1275	-4.70	1.0e-10	5.2e-8

Significant changes are defined as *p*-value < 0.001, FDR < 0.01, and Log2FC > 1.5. Abbreviations: FC, fold change; FDR, false discovery rate

### Validation study for miRNAs expression

A total of 69 individuals were analysed in the second step of the study (40 subjects with COVID-19 and 29 non-COVID-19 individuals as controls). Within cases, all of them presented mild symptoms (fever, headache, cough, etc.).

The average age of the study cohort was 43 years (± 13), comparable between cases and controls (*p* = 0.101; 41 and 46 years for cases and the control group, respectively). Additionally, cases and controls were similar concerning sex distribution (*p* = 0.684), with 47.1% of cases and 50% of controls being male.

### miRNAs expression analysis

**Fig. 3:**
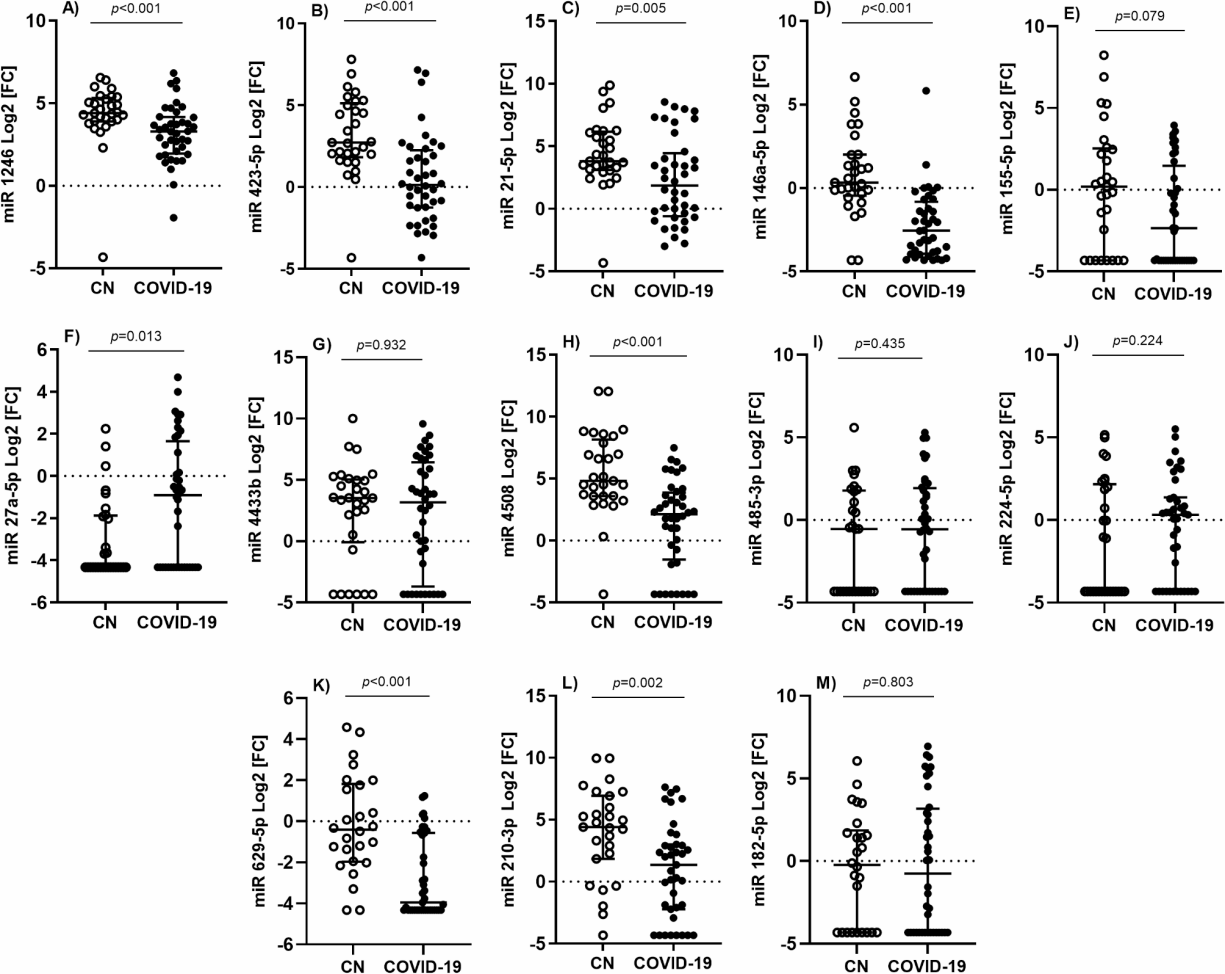
Differential miRNA expression in individuals with COVID-19 in contrast to control subjects without infection (CN). Lines represent the median with an interquartile range. *p*-values < 0.05 were considered significant

The 11 most dysregulated miRNAs (from the NGS screening), plus the four selected ones from outstanding referenced studies, were analysed in the validation step. From them, seven miRNAs were found to be downregulated in individuals with COVID-19 (Fig. 3). Only miR-27a-5p was overexpressed in infected individuals. Moreover, the expression of miR-1246, miR-423-5p, miR-21-5p, miR-146a-5p, miR-4508, miR-629-5p and miR-210-3p was associated with non-infectious state, whereas the expression of miR-27a-5p was associated with SARS-CoV-2 infection (Table 2). miR-369-5p and miR-143-5p gene expression was observed in less than 10% of the samples under study, so they were excluded for further analysis.

Concerning clinical features, none of the altered miRNA’s expression was significantly correlated with age, without significant differences between sexes.

**Table 2:** Binary logistic regression analysis showing the adjusted effect of differential expression of studied miRNAs

miRNA	OR	CI (95%)	*p*-value
miR-1246	0.667	0.470–0.945	0.023
miR-423-5p	0.678	0.539–0.852	< 0.001
miR-21-5p	0.809	0.684–0.956	0.013
miR-146a-5p	0.551	0.404–0.753	< 0.001
miR-155-5p	-	-	ns
miR-27a-5p	1.327	1.069–1.647	0.010
miR-4433b-5p	-	-	ns
miR-4508	0.697	0.567–0.855	< 0.001
miR-485-3p	-	-	ns
miR-224-5p	-	-	ns
miR-629-5p	0.598	0.451–0.792	< 0.001
miR-210-3p	0.818	0.708–0.946	0.007
miR-182-5p	-	-	ns

### Functional annotation analysis and gene target prediction

The in-silico functional analysis, through the KEGG pathways, revealed the resulting altered miRNAs in mild COVID-19 to be involved in different biological processes (Supplementary Table 2). miR-1246 was involved in viral carcinogenesis. miRNAs related to immune processes were also found, miR-146a-5p was involved in the toll-like receptor signalling pathway and miR-27a-5p in the TGF-beta signalling pathway. Moreover, miR-21-5p, miR-27a-5p and miR-210-3p were involved in cancer pathways.

Moreover, the GO analysis was enriched in several functions (Supplementary Table 3). miR-423-5p was involved in modification by virus-host mRNA processing (*p* = 0.038), viral protein processing (*p* = 0.018) and the viral cell life (*p* = 0.001). miR-4508 was involved in the viral protein processing (*p* = 0.040). We also found that miR-423-5p was involved in the apoptotic signalling pathway (*p* = 0.003), miR-27a-5p in the positive regulation of the apoptotic process (*p* = 0.049) and miR-21-5p in the negative regulation of apoptosis (*p* = 0.037). Respecting the immune response, miR-423-5p (*p* = 0.004), miR-21-5p (*p* = 0.006) and miR-27a-5p (*p* = 0.041) were involved in the leukocyte migration. Additionally, miR-21-5p (*p* = 0.0001) and miR-146a-5p (*p* = 0.003) had a role in the toll-like receptor signalling pathways, while miR-21-5p was involved in the transforming growth factor receptor signalling (*p* = 0.005). The in-silico analysis predicted the involvement of miR-423-5p (*p* = 1.79e-09) and miR-21-5p (*p* = 2.42e-12) in response to stress.

## Discussion

miRNAs are considered potential biomarkers for multiple diseases. It is well-known that viruses, like SARS-CoV-2, can alter miRNA expression for their benefit. What is more, host cells regulate miRNAs to fight against the infection. The identification of biomarkers can help identify individuals at higher risk for developing severe forms of the disease, thus enhancing patient prognosis. This study describes the expression patterns of 13 dysregulated miRNAs in serum samples from mild COVID-19 cases in contrast to a non-infected control group.

Seven miRNAs were downregulated in COVID-19 cases while only miR-27a-5p was overexpressed in infected individuals. The decreased expression of miR-27a-5p has been related to pneumonia and bilateral interstitial lesions in COVID-19 patients with several cardiovascular comorbidities (Pieri et al. 2023). The inhibition of this miRNA is also related to pulmonary fibrosis, modulating the TGF-beta pathway (Córdoba-Lanús et al. 2023; Kang 2017). The KEGG analysis revealed enriched pathways in TGF-beta signalling. The modulation of the expression of certain genes, like *SMAD2* and *E2F4*, in the TGF-beta pathway can induce apoptosis. Interestingly, we found through the in-silico analysis that miR-27a-5p was involved in the positive regulation of the apoptotic process. In our study, the higher expression of miR-27a-5p in mild COVID-19 cases may be explained by a protective effect against severe forms of the disease.

From the resulting seven downregulated miRNAs, miR-1246 is particularly interesting. The KEGG pathway analysis revealed that miR-1246 is involved in viral carcinogenesis, acting on *BAX*,* CDK6*,* TP53* and *CCN1*. These genes are involved in the p53 signalling pathway, a well-known pathway in cancer. The p53 protein is involved in cell differentiation, cell cycle, and apoptosis (Ghafouri-Fard et al. 2022; Wang et al. 2023). Furthermore, p53 is involved in the antiviral defence of the host cells. Viral replication is enhanced in cells that lack p53, while in the cells that express p53, the opposite effect is observed (Ramaiah 2020). The study carried out by Wu et al., found higher levels of miR-1246 in plasma samples from patients during the acute phase of COVID-19 (Wu et al. 2021). In our study, the downregulation of this miRNA in mild COVID-19 cases supposes that the p53 pathway is not blocked, conducing to the correct control of the infection.

miR-423-5p has been extensively studied regarding cancer progression and treatment response (Shan et al. 2020; Stiuso et al. 2015; Yang et al. 2018). In patients with adenocarcinoma, miR-423-5p inhibits *CADM1* expression, aggravating the progression of the cancer(Huang and Feng 2021). In COPD patients, the miR-423-5p expression was decreased, suggesting its use as a potential diagnostic biomarker for the disease(Zhang et al. 2022). The downregulation of miR-423-5p in mild COVID-19 cases found in our study is in concordance with Farr et.al., who found increased levels of this miRNA in hospitalized patients(Farr et al. 2021). miR-423-5p seems a suitable biomarker for COVID-19 in the early stages of the infection.

Concerning other miRNAs, miR-629-5p expression has been described to enhance cancer invasion by altering the endothelial cell permeability (Li et al. 2020). This miRNA was reported to be upregulated in hospitalized COVID-19 patients (Franco et al., 2024). In our study, decreased levels of miR-629-5p were found in mild cases, suggesting that the upregulation of this miRNA might be characteristic of severe forms of the disease. Another miRNA known to promote lung carcinoma is miR-210-5p, whose expression is induced by hypoxia(Arora et al. 2023). The expression of this miRNA has been related to the inhibition of *E2F3* and the progression of lung adenocarcinoma (Zhang et al. 2023). In our study, the KEGG analysis showed an interaction with *E2F3*, suggesting that the absence of low oxygen saturation in mild COVID-19 cases may account for the reduced expression of miR-210-3p. An in-silico study indicated that miR-210-3p may have an important role in the pathogenesis of COVID-19 by regulating the adaptive response to hypoxia (Baig et al. 2023).

The study by Guiot et al. has demonstrated that miR-21-5p induces fibrosis by interfering with *SMAD7* (Guiot et al. 2023). The KEGG analysis reflected that this miRNA acts on *SMAD7* in the Hippo signalling pathway. This pathway regulates the inflammatory response in the lungs, and its activation contributes to pulmonary inflammation and the progression of fibrosis (Fu et al. 2022). Related to COVID-19, an increased expression of miR-21-5p has been found in severe cases (Giannella et al. 2022). Moreover, a recent study of COVID-19 patients under 55 years, revealed an association between the expression of miR-21-5p and severity and mortality (Bautista-Becerril et al. 2023). In our research, the downregulation of miR-21-5p observed in mild COVID-19 cases confirms that the increase in the expression of this miRNA contributes to severe forms of the disease. Concerning the dysregulation of miR-146a-5p expression, higher levels of this miRNA were observed in COVID-19 patients with worse outcomes (Bautista-Becerril et al. 2023; Giannella et al. 2022). We found low levels of miR-146a-5p in infected mild individuals, confirming that high levels of miR-146a-5p may be characteristic of severe COVID-19 cases. miR-146a-5p plays a role in the toll-like receptor signalling pathway, critical for pathogen recognition and innate immune system stimulation (Akira and Takeda 2004).

miR-4508 was another one of the miRNAs that we found downregulated in mild cases. The KEGG analysis revealed that this miRNA is involved in cell adhesion molecules essential for immune-cell interaction and migration through tissues (Harjunpää et al. 2019). miR-4508 acts on two genes related to this process, *ICAM2* and *CLDN5*. A shortage of ICAM2 protein expression has been associated with worse outcomes of COVID-19 (Zhu et al., 2021). Furthermore, SARS-CoV-2 promotes the disruption of the endothelial barrier in the lungs by inhibiting the expression of *CLDN5 *(Hashimoto et al. 2022). The downregulation of miR-4508 avoids the inhibition of these genes, promoting the efficient control of the infection.

This study has limitations. First, the present study focused on mild COVID-19 cases, excluding severe cases which might be necessary to confirm some of the present findings. Second, miRNA expression can vary between different types of tissues, which may occur in inflammatory cells and lymphoid organs during the immune response, as opposed to serum. Third, further functional in-vitro assays should be necessary to confirm our findings. Lastly, replication on an independent cohort is needed to endorse our results.

## Conclusions

In conclusion, miR-27a-5p expression is related to a protective effect against severe forms of COVID-19. The downregulation of miR-1246 has been associated with an efficient antiviral cell host defence while decreased miR-21-5p expression may prevent the development of pulmonary fibrosis. Moreover, miR-423-5p is suggested as a useful biomarker in the early stages of SARS-CoV-2 infection. Future prospective studies in a larger cohort are needed to confirm present findings.

## Electronic supplementary material

Below is the link to the electronic supplementary material.

**Supplementary Material 1: Table 1.** Selected assays for the analysis of the miRNAs included in the study

**Supplementary Material 2: Table 2.** Principal enriched KEGG pathways of predicted target genes of the analysed miRNAs

**Supplementary Material 3: Table 3.** Targeted enriched Gene-ontology (GO) biological processes by the analysed miRNAs

**Supplementary Material 4**

## Data Availability

Data supporting the statistics from experimental phase as well as graphic material are available upon reasonable request. Data from Next Generation Sequencing and data obtained from in-silico analysis has been added as supplementary material.
