# Supplementary material for: miR-27a-5p, miR-21-5p, miR-1246 and miR-4508: a candidate microRNA signature in the protection and regulation of viral infection in mild COVID-19
Source: Mol Med. 2025 Mar 15;31:102. doi: 10.1186/s10020-025-01154-0 (PMC11910857; doi:10.1186/s10020-025-01154-0)
Supplement: Supplementary file 1 — Supplementary Material 1: Table 1. Selected assays for the analysis of the miRNAs included in the study [file 10020_2025_1154_MOESM1_ESM.docx]

**Supplementary Table 1**. Selected assays for the miRNAs included in the study.

| **Name** | **Gen Globe ID** | **Accession** | **Sequence** |
| --- | --- | --- | --- |
| hsa-miR-26a-5p | YP00206023 | MIMAT0000082 | 5'UUCAAGUAAUCCAGGAUAGGCU |
| hsa-miR-1246 | YP00205630 | MIMAT0005898 | 5'AAUGGAUUUUUGGAGCAGG |
| has-miR-27a-5p | YP00206021 | MIMAT0004501 | 5'AGGGCUUAGCUGCUUGUGAGCA |
| has-miR-4433b-5p | YP02118899 | MIMAT0030413 | 5'AUGUCCCACCCCCACUCCUGU |
| has-miR-4508 | YP02101084 | MIMAT0019045 | 5'GCGGGGCUGGGCGCGCG |
| has-miR-485-3p | YP00206055 | MIMAT0002176 | 5'GUCAUACACGGCUCUCCUCUCU |
| has-miR-224-5p | YP02119313 | MIMAT0000281 | 5'UCAAGUCACUAGUGGUUCCGUUUAG |
| has-miR-629-5p | YP00204370 | MIMAT0004810 | 5'UGGGUUUACGUUGGGAGAACU |
| has-miR-369-5p | YP00206014 | MIMAT0001621 | 5'AGAUCGACCGUGUUAUAUUCGC |
| has-miR-155-5p | YP02119311 | MIMAT0000646 | 5'UUAAUGCUAAUCGUGAUAGGGGUU |
| hsa-miR-21-5p | YP00204230 | MIMAT0000076 | 5'UAGCUUAUCAGACUGAUGUUGA |
| hsa-miR-146a-5p | YP00204688 | MIMAT0000449 | 5'UGAGAACUGAAUUCCAUGGGUU |
| hsa-miR-210-3p | YP00204333 | MIMAT0000267 | 5'CUGUGCGUGUGACAGCGGCUGA |
| hsa-miR-182-5p | YP00206070 | MIMAT0000259 | 5'UUUGGCAAUGGUAGAACUCACACU |
