# Supplementary material for: miR-27a-5p, miR-21-5p, miR-1246 and miR-4508: a candidate microRNA signature in the protection and regulation of viral infection in mild COVID-19
Source: Mol Med. 2025 Mar 15;31:102. doi: 10.1186/s10020-025-01154-0 (PMC11910857; doi:10.1186/s10020-025-01154-0)
Supplement: Supplementary file 2 — Supplementary Material 2: Table 2. Principal enriched KEGG pathways of predicted target genes of the analysed miRNAs [file 10020_2025_1154_MOESM2_ESM.docx]

**Supplementary Table 2**. Principal enriched KEGG pathways of predicted target genes of the analysed miRNAs.

| **#** | **KEGG pathway** | **Genes** | **p-value** |
| --- | --- | --- | --- |
| **miR-1246** | | | |
| 1 | Viral carcinogenesis | PIK3CB, BAX, CDK6, TP53, KAT2B, CCNE1. | 1.8165857589e-05 |
| 2 | Oocyte meiosis | SLK, SMC1A, PPP2CA, CPEB4, CALM2, PPP3CA, CCNE1, PPP2R1B | 0.00536006388815 |
| 3 | Apoptosis | CASP7, PIK3CB, BAX, BCL2, TP53, PPP3CA, CFLAR | 0.00718280448455 |
| 4 | Thyroid hormone signaling pathway | MED14, PIK3CB, MED13, NOTCH2, TP53, MED1, KAT2B | 0.0211813727917 |
| 5 | Central carbon metabolism in cancer | PIK3CB, TP53, PDK1 | 0.0211813727917 |
| 6 | p53 signaling pathway | BAX, CDK6, TP53, TP53I3, CCNE1, CCNG2 | 0.0448382131831 |
| 7 | Adrenergic signaling in cardiomyocytes | PIK3CB, PPP2CA, BCL2, CALM2, ATP2B1, PPP2R1B | 0.0448382131831 |
| 8 | Glioma | PIK3CB, CDK6, CALM2, TP53 | 0.0448382131831 |
| **miR-423-5p** | | | |
| 1 | Fatty acid biosynthesis | FASN, ACSL3 | 7.10099401607e-17 |
| 2 | Lysine degradation | WHSC1L1, SETD7, SETD1B, NSD1, ASH1L, OGDH, SETD8, KMT2D, DOT1L, SUV420H2, KMT2A, KMT2C, SETD1A | 7.09607295278e-09 |
| 3 | Biosynthesis of unsaturated fatty acids | FADS1, ACOT2, PTPLA, SCD, TECR | 5.30062382268e-08 |
| 4 | Fatty acid metabolism | FASN, ACSL3, FADS1, PTPLA, SCD, TECR | 6.76558435038e-07 |
| 5 | N-Glycan biosynthesis | RPN1, DOLPP1, DPM2, B4GALT1, ALG10B, ALG14, MAN1B1, DDOST, DOLK, B4GALT3, MGAT1, ALG3, MGAT4B | 0.00679744182654 |
| 6 | Glycosaminoglycan biosynthesis - keratan sulfate | B3GNT7, ST3GAL1, B4GALT1, B4GALT3 | 0.0105163177967 |
| 7 | Huntington's disease | POLR2B, HTT, IFT57, TAF4, ATP5B, CREB1, PLCB1, DNAH9, ATP5G3, POLR2A, POLR2H, NDUFS1, NDUFB7, POLR2E, HDAC2, DNALI1, CASP9, SP1, EP300, BBC3, CASP8, NDUFA13, CREB3L2, SOD2, AP2M1, MT-CO1, MT-ATP6 | 0.0105163177967 |
| 8 | Adherens junction | ACTB, RAC2, SMAD2, ACTG1, LMO7, IGF1R, PTPRF, MLLT4, WASF2, SRC, MAPK3, ACTN4, EP300, LEF1, PTPRB, MAP3K7 | 0.0131881576434 |
| 9 | Estrogen signaling pathway | GABBR1, CALML5, CALM3, CALM1, GNAS, SHC1, FKBP4, HSPA1L, RAF1, CREB1, PLCB1, MMP2, GRM1, PRKACA, SOS1, SRC, MAPK3, SP1, CREB3L2, GNAI2, GRB2, ADCY6 | 0.0131881576434 |
| 10 | Bacterial invasion of epithelial cells | ARPC5, ACTB, CRKL, CRK, SHC1, SEPT11, ACTG1, ITGA5, DOCK1, WASF2, SRC, FN1, SEPT9, RHOG, ILK | 0.0131881576434 |
| **miR-21-5p** | | | |
| 1 | Fatty acid elongation | PTPLB, HADH, HADHA, ACOT7, ELOVL7, ELOVL4 | 1.52100912653e-05 |
| 2 | Lysine degradation | WHSC1L1, SETD1B, HADH, ASH1L, HADHA, KMT2D, SUV420H1, WHSC1, KMT2A, ACAT1, KMT2C, COLGALT1 | 6.28342857219e-05 |
| 3 | Proteoglycans in cancer | ESR1, ACTB, STAT3, PDCD4, EZR, SOS2, NRAS, THBS1, WNT5A, ARHGEF12, ROCK2, FRS2, IQGAP1, TIAM1, IGF1R, TLR4, FASLG, CTTN, PTK2, CCND1, CTNNB1, MYC, MMP9, TIMP3, PIK3R1, FAS, TGFB2, GAB1, SDC2, VEGFA, MAPK1, MDM2, CD44 | 8.01642798876e-05 |
| 4 | Colorectal cancer | TGFBR1, APC, BIRC5, AXIN1, APPL1, CCND1, CTNNB1, MYC, MSH2, PIK3R1, TGFB2, MAPK1, TGFBR2 | 0.00208666389186 |
| 5 | Pathways in cancer | STAT3, TGFBR1, SOS2, LAMB1, NRAS, CRKL, BID, APC, CUL2, WNT5A, ARHGEF12, ROCK2, GNG12, RALA, BIRC5, PLD1, AXIN1, IGF1R, APPL1, FASLG, PTK2, CCND1, CTNNB1, RASGRP1, SKP2, E2F3, F2R, MYC, MMP9, MSH2, HSP90AB1, VEGFC, PIK3R1, HSP90B1, HDAC2, FGF18, FAS, LAMC1, TGFB2, GNAQ, SLC2A1, RASGRP3, VEGFA, PTEN, MAPK1, TGFBR2, MDM2, CCDC6, EGLN1, COL4A1, RXRA, NCOA4 | 0.00208666389186 |
| 6 | Thyroid hormone signaling pathway | ESR1, ACTB, NRAS, MED13, SLC16A10, ATP1B1, NCOA3, CCND1, CTNNB1, MYC, NCOR1, NCOA2, PIK3R1, HDAC2, THRB, SLC2A1, PFKFB2, MAPK1, MDM2, RXRA | 0.00578984100127 |
| 7 | Fatty acid metabolism | PTPLB, HADH, HADHA, SCD, ACSL4, ACAT1, ACSL6 | 0.0072067771321 |
| 8 | Fatty acid degradation | HADH, ECI2, HADHA, ACSL4, ACAT1, ACSL6 | 0.00941890914992 |
| 9 | FoxO signaling pathway | STAT3, TGFBR1, SOS2, NRAS, PRKAA2, CCND2, IGF1R, FASLG, CCND1, SKP2, PIK3R1, BCL6, PRKAB2, TGFB2, SOD2, FOXO3, USP7, PLK1, PTEN, SGK3, MAPK1, IL10, CCNG2, TGFBR2, MDM2 | 0.00941890914992 |
| 10 | Hippo signaling pathway | ACTB, TGFBR1, YAP1, BTRC, APC, NF2, WNT5A, DLG1, CCND2, BIRC5, AXIN1, MPP5, CCND1, CTNNB1, MYC, SAV1, TGFB2, SMAD7, LATS1, SOX2, PPP2R1B, TGFBR2, BMPR2 | 0.0114551181946 |
| **miR-146a-5p** | | | |
| 1 | Thyroid hormone synthesis | TTF2, GPX6, LRP2, TG, IYD | 0.000237855879605 |
| 2 | ErbB signaling pathway | CAMK2D, BRAF, GSK3B, NRAS, EGFR, STAT5B, AKT2, JUN, CDKN1A, ABL2, ABL1, ERBB4 | 0.000281568508137 |
| 3 | Adherens junction | WASL, BAIAP2, IQGAP1, EGFR, FYN, SMAD4, ACTN1, FER, YES1, MAP3K7 | 0.0149880505022 |
| 4 | Toll-like receptor signaling pathway | TLR2, CD80, TAB2, AKT2, JUN, TRAF6, STAT1, IRF7, IRAK1, MAP3K7 | 0.0476478069913 |
| **miR-155-5p** | | | |
| 1 | Hepatitis B | FOS, STAT3, NFKB1, CDK4, E2F2, CDK2, SMAD3, BCL2, DDB2, KRAS, CREB1, APAF1, MAVS, MYD88, CCND1, SMAD4, E2F3, PIK3R1, YWHAZ, TBK1, AKT3, PIK3CA, CDKN1A, STAT1, TNF, RELA, IL6, MAPK10 | 2.36088260682e-06 |
| 2 | Arrhythmogenic right ventricular cardiomyopathy (ARVC) | CDH2, ITGB1, CACNB4, TCF7L2, ITGB4, ITGB5, PKP2, DMD, CTNNB1, CTNNA1, SLC8A1, DAG1, DSG2, JUP | 1.88383080888e-05 |
| 3 | TGF-beta signaling pathway | SMAD2, THBS1, PPP2CA, SMAD3, SMAD4, SMAD5, ACVR2A, GDF6, SP1, ACVR1C, PPP2CB, TNF, SMAD1, RPS6KB1 | 4.57959931122e-05 |
| 4 | FoxO signaling pathway | IRS2, STAT3, SMAD2, SETD7, MAPK14, CDK2, PCK2, SMAD3, CAT, EGFR, KRAS, CCND1, SMAD4, S1PR1, GABARAPL1, PIK3R1, SOS1, AKT3, PIK3CA, FOXO3, USP7, CDKN1A, PLK1, SGK3, FOXO1, IL6, IL10, MDM2, MAPK10, C8orf44-SGK3 | 0.000136124980428 |
| 5 | Pancreatic cancer | STAT3, NFKB1, CDK4, SMAD2, E2F2, SMAD3, EGFR, CDKN2A, ARHGEF6, KRAS, CCND1, SMAD4, E2F3, PIK3R1, AKT3, PIK3CA, STAT1, RELA, MAPK10 | 0.000418505229032 |
| 6 | Apoptosis | NFKB1, IL1B, BCL2, PRKAR1B, DFFA, APAF1, MYD88, MAP3K14, TNFRSF10A, PIK3R1, CFLAR, PRKAR2A, TNFRSF10B, PRKAR1A, AKT3, PIK3CA, BIRC3, AIFM1, TNF, RELA, XIAP | 0.00151497018364 |
| 7 | Prolactin signaling pathway | FOS, GSK3B, STAT3, NFKB1, SHC1, MAPK14, KRAS, SOCS6, CCND1, SOCS1, SOCS5, PIK3R1, SOS1, AKT3, PIK3CA, FOXO3, STAT1, RELA, MAPK10 | 0.00349286651991 |
| 8 | Steroid biosynthesis | DHCR24, HSD17B7, CYP51A1, FDFT1 | 0.00409660012621 |
| 9 | TNF signaling pathway | FOS, NFKB1, IL1B, MAPK14, VCAM1, TAB2, RPS6KA5, ICAM1, CREB1, CEBPB, MAP3K14, JUNB, PIK3R1, CFLAR, AKT3, PIK3CA, BIRC3, TNF, RELA, IL6, TRAF3, MAPK10 | 0.00409660012621 |
| 10 | Signaling pathways regulating pluripotency of stem cells | JARID2, GSK3B, STAT3, FZD5, KAT6A, SMAD2, WNT5A, MAPK14, SMAD3, KRAS, POU5F1B, PCGF5, RIF1, SMAD4, CTNNB1, ZIC3, SMAD5, PIK3R1, ACVR2A, FGF2, ACVR1C, AKT3, PIK3CA, IL6ST, SMAD1, MEIS1 | 0.00409660012621 |
| **miR-27a-5p** | | | |
| 1 | Signaling pathways regulating pluripotency of stem cells | APC, WNT4, IGF1R, ZFHX3, FZD4, SMAD4, CTNNB1, AKT1, SMAD5, MAPK3, BMP2, ACVR1C, BMPR1A, GRB2, SMAD1, DVL2 | 1,23E-05 |
| 2 | Proteoglycans in cancer | PDCD4, THBS1, ARHGEF12, WNT4, IGF1R, RHOA, RPS6, FZD4, MMP2, PLCG1, GPC1, CTNNB1, AKT1, IGF2, FLNB, MAPK3, FN1, GRB2, MDM2 | 1,31E-05 |
| 3 | Fatty acid elongation | HADHA | 6,43E-05 |
| 4 | ECM-receptor interaction | THBS1, COL4A2, LAMC1, FN1, ITGA4, SPP1 | 9,98E-05 |
| 5 | Glioma | PDGFRA, CALM3, SHC1, IGF1R, CALM2, PLCG1, AKT1, MAPK3, GRB2, MDM2 | 1,91E-04 |
| 6 | TGF-beta signaling pathway | THBS1, RHOA, SMAD4, SMAD5, MAPK3, BMP2, ACVR1C, BMPR1A, LTBP1, E2F4, SMAD1 | 0.000500356725655 |
| 7 | Hippo signaling pathway | BTRC, APC, NF2, WNT4, WWTR1, FZD4, SMAD4, CTNNB1, CTNNA1, BMP2, BMPR1A, SMAD1, DVL2 | 0,000543478 |
| 8 | Wnt signaling pathway | LRP6, BTRC, APC, DKK2, CTBP2, WNT4, RHOA, NFATC4, NLK, FZD4, SMAD4, CTNNB1, CSNK1A1, SFRP1, FOSL1, DVL2, PPARD | 0.000643872390825 |
| 9 | Adherens junction | PTPRM, PTPN1, IGF1R, RHOA, NLK, SMAD4, CTNNB1, CTNNA1, FER, MAPK3, ACTN4 | 0,00135034 |
| 10 | Pathways in cancer | PDGFRA, APC, ARHGEF12, BCR, CTBP2, RALBP1, WNT4, IGF1R, RHOA, VHL, EPAS1, FZD4, MMP2, AR, PLCG1, SMAD4, CTNNB1, CTNNA1, COL4A2, AKT1, HSP90AB1, MAPK3, BMP2, LAMC1, PPARG, FN1, RALB, GRB2, DVL2, TFG, PPARD, MDM2, ABL1 | 0,002126629 |
| miR-4433b-5p | | | |
| 1 | Arachidonic acid metabolism | CYP2J2 | 0.000829144857534 |
| 1 | Glycosylphosphatidylinositol(GPI)-anchor biosynthesis | PIGM, PIGS | 0.0293927703161 |
| 2 | Bile secretion | CFTR, SLC10A2, ABCB11 | 0.0293927703161 |
| 3 | Proteoglycans in cancer | CD44, PPP1CB | 0.0293927703161 |
| **miR-4508** | | | |
| 1 | Fatty acid elongation | HADHA | 1.70968709218e-12 |
| 2 | Fatty acid degradation | HADHA | 7.27315281045e-08 |
| 3 | Fatty acid metabolism | HADHA | 7.27315281045e-08 |
| 4 | Cell adhesion molecules (CAMs) | \| ICAM2, CLDN5, PVRL1 \| \| --- \| | 0.000122113171084 |
| 5 | Glycosphingolipid biosynthesis ganglio series | ST8SIA5 | 0.000239624831233 |
| 6 | Nicotine adiction | CACNA1A, GRIN2D | 0.0002396248312332 |
| 7 | Metabolism of xenobiotics by cytochrome p450 | GSTM1 | 0.00115532088992 |
| 8 | Lysine degradation | HADHA, DOT1L, SUV420H2 | 0.0034296575703 |
| 9 | Primary bile acid biosynthesis | SLC27A5 | 0.0282712109655 |
| **miR-485-3p** | | | |
| 1 | Axon guidance | PPP3R1, GNAI3, RHOA, PTK2, DCC, NRP1, RAC1, UNC5D | 0.000112696679027 |
| 2 | Adherens junction | SNAI2, RHOA, RAC1, CTNNA3, PVRL3 | 0.0106273984145 |
| 3 | Leukocyte transendothelial migration | PRKCA, GNAI3, RHOA, ARHGAP35, PTK2, RAC1, CTNNA3, VAV3, RAP1B | 0.0382192049159 |
| **miR-224-5p** | | | |
| 1 | Lysine degradation | CAMKMT, WHSC1L1, SETD7, SETD2, NSD1, ASH1L, HADHA, SETDB1, AADAT, KMT2D, DOT1L, SUV420H2, ALDH9A1 | 1.43712346163e-10 |
| 2 | Glioma | PDGFRA, E2F1, E2F2, NRAS, CALM3, CALM1, PIK3CB, IGF1R, EGFR, CDK6, PIK3R3, CCND1, PIK3CG, CDKN1A | 0.000212814635074 |
| 3 | Melanoma | PDGFRA, E2F1, E2F2, NRAS, PIK3CB, IGF1R, EGFR, CDK6, MITF, CDH1, PIK3R3, CCND1, PIK3CG, CDKN1A, HGF | 0.00614832611878 |
| 4 | Fatty acid elongation | HADHA, ELOVL5 | 0.00659373036855 |
| 5 | Hippo signaling pathway | ACTB, GSK3B, PRKCI, DLG1, BMPR1B, YWHAG, PPP1CC, GLI2, FZD6, MPP5, CDH1, CCND1, SMAD4, YWHAZ, WTIP, LATS1, PPP2R1B, SERPINE1, PARD6B, WNT9A | 0.00659373036855 |
| 6 | Fatty acid metabolism | ACSL3, PECR, HADHA, ELOVL5, SCD, ACSL4, ACACA | 0.00711782519825 |
| 7 | Biosynthesis of unsaturated fatty acids | PECR, HADHA, ELOVL5, SCD | 0.00832998639192 |
| 8 | Viral carcinogenesis | H2BFM, ATF2, NRAS, H2BFWT, PIK3CB, DLG1, YWHAG, HLA-E, CDKN1B, CDK6, DDX3X, CHEK1, PIK3R3, CCND1, EGR2, REL, ACTN1, PIK3CG, YWHAZ, IL6ST, CDKN1A, HLA-A, UBR4, HIST1H4E | 0.00908269259115 |
| 9 | Proteoglycans in cancer | ACTB, CBL, NRAS, PIK3CB, ARHGEF12, PPP1CC, MAPK14, FZD6, IGF1R, EGFR, ANK3, DCN, PIK3R3, CCND1, IGF2, FLNB, PIK3CG, MAPK12, HOXD10, FN1, CDKN1A, SDC4, HGF, WNT9A | 0.0162451103992 |
| 10 | Prolactin signaling pathway | PRLR, GSK3B, SOCS4, NRAS, PIK3CB, MAPK14, PIK3R3, CCND1, MAPK8, PIK3CG, MAPK12 | 0.0191668936714 |
| **miR-629-5p** | | | |
| 1 | Steroid biosynthesis | DHCR24 | 0,00010973 |
| **miR-210-3p** | | | |
| 1 | Other types of O-glycan biosynthesis | ST3GAL3 | 0,000899217 |
| 2 | MicroRNAs in cancer | KIF23, E2F3, RASSF1 | 0,006278296 |
| 3 | Pancreatic secretion | CHRM3, RASB27B, ATP2B3 | 0,037894849 |
| **miR-182-5p** | | | |
| 1 | Fatty acid biosynthesis | FASN, ACSL4, ACACA | 3.27117132347e-11 |
| 2 | Viral carcinogenesis | STAT3, NFKB1, CDK4, NRAS, RASA2, YWHAE, HDAC3, HIST1H2BK, PIK3CB, CREB5, SYK, YWHAG, CCND2, BAX, PKM, CDKN1B, CDK1, CDK6, STAT5B, DDX3X, HIST1H2BD, HDAC9, TP53, EGR3, CREB1, EIF2AK2, SND1, CCND1, SKP2, TBPL2, DDB1, HIST1H4H, PIK3R1, RB1, YWHAZ, RAC1, CDC42, EP300, CREB3L2, CCNE1, PIK3CA, CREB3L1, BAK1, CDKN1A, TBP, RELA, GTF2A1, SRF, CREBBP, GRB2, MAPKAPK2, ATF4, PRKACB, HIST1H4I | 4.00333527532e-09 |
| 3 | Adherens junction | CSNK2A2, MET, WASF1, WASL, CTNND1, PVRL2, TCF7L2, IQGAP1, SNAI2, PTPN1, IGF1R, VCL, WASF3, TJP1, FYN, SMAD4, CTNNB1, CTNNA1, WASF2, CSNK2A1, RAC1, CDC42, SSX2IP, EP300, CTNNA3, YES1, CREBBP, PVRL1 | 6.62266658689e-07 |
| 4 | Estrogen signaling pathway | ESR1, HBEGF, NRAS, ADCY2, CALM1, SHC1, PIK3CB, CREB5, CREB1, AKT1, FKBP5, ITPR1, PIK3R1, SOS1, KCNJ6, HSPA8, SP1, SHC4, GNAQ, AKT3, CREB3L2, GNAI2, PIK3CA, CREB3L1, ITPR3, GRB2, KCNJ5, PLCB4, ATF4, PRKACB, ADCY6 | 8.68093254951e-06 |
| 5 | Hepatitis B | STAT3, E2F1, NFKB1, CDK4, NRAS, PIK3CB, CREB5, BAX, BCL2, CDKN1B, BIRC5, MAP3K1, CDK6, STAT5B, DDX3X, TP53, EGR3, CREB1, MAVS, CCND1, SMAD4, MAPK8, AKT1, DDB1, PIK3R1, RB1, YWHAZ, PRKCB, EP300, AKT3, CREB3L2, CCNE1, PIK3CA, CREB3L1, CDKN1A, MAP2K4, RELA, NFATC3, CREBBP, GRB2, ELK1, ATF4 |  |
| 6 | Glioma | BRAF, PDGFRA, E2F1, CDK4, NRAS, CALM1, SHC1, PIK3CB, IGF1R, CDK6, TP53, PLCG1, CCND1, AKT1, PIK3R1, RB1, SOS1, PRKCB, SHC4, AKT3, PIK3CA, CDKN1A, GRB2 | 0.000110072858072 |
| 7 | Prostate cancer | BRAF, GSK3B, PDGFRA, E2F1, NFKB1, NRAS, PIK3CB, CREB5, TCF7L2, BCL2, CDKN1B, IGF1R, TP53, CREB1, CCND1, CTNNB1, AKT1, PIK3R1, RB1, SOS1, EP300, AKT3, CREB3L2, CCNE1, PDGFD, PIK3CA, CREB3L1, CDKN1A, PDPK1, RELA, FOXO1, CREBBP, GRB2, ATF4 | 0.000158413240059 |
| 8 | Bacterial invasion of epithelial cells | MET, WASF1, ITGB1, WASL, CBL, DNM2, SHC1, PIK3CB, ARPC3, CLTC, VCL, CD2AP, CTTN, PTK2, CTNNB1, CTNNA1, WASF2, PIK3R1, RAC1, CDC42, SHC4, ELMO1, CTNNA3, PIK3CA, SEPT6, ARPC4, SEPT9, RHOG | 0.000319136166785 |
| 9 | SNARE interactions in vesicular transport | SNAP23, STX5, STX10, STX3, VAMP1, GOSR2, STX7, VTI1B, STX6, STX2, VAMP2, SEC22B, VAMP3 | 0.000777886416861 |
| 10 | Chronic myeloid leukemia | BRAF, E2F1, NFKB1, CDK4, CBL, NRAS, SHC1, PIK3CB, CDKN1B, CDK6, STAT5B, TP53, CCND1, SMAD4, AKT1, PIK3R1, RB1, SOS1, SHC4, BCL2L1, AKT3, PIK3CA, MECOM, CDKN1A, RELA, GRB2 | 0.000777886416861 |
