# Supplementary material for: miR-27a-5p, miR-21-5p, miR-1246 and miR-4508: a candidate microRNA signature in the protection and regulation of viral infection in mild COVID-19
Source: Mol Med. 2025 Mar 15;31:102. doi: 10.1186/s10020-025-01154-0 (PMC11910857; doi:10.1186/s10020-025-01154-0)
Supplement: Supplementary file 3 — Supplementary Material 3: Table 3. Targeted enriched Gene-ontology (GO) biological processes by the analysed miRNAs [file 10020_2025_1154_MOESM3_ESM.docx]

**Supplementary Table 3.** Targeted enriched Gene-ontology (GO) biological processes by the analysed miRNAs.

| **#** | **Go category** | **Genes** | **p-value** |
| --- | --- | --- | --- |
| **miR-1246** | | | |
| 1 | Cellular nitrogen compound metabolic process | 204 | 2.28790724676e-18 |
| 2 | Biosynthetic process | 161 | 8.83162305156e-10 |
| 3 | Cellular protein modification process | 103 | 2.21979703119e-08 |
| 4 | Gene expression | 31 | 1.45841773627e-05 |
| 5 | Viral process | 24 | 0.0011158114917 |
| 6 | Symbiosis, encompassing mutualism through parasitism | 25 | 0.00321424260912 |
| 7 | Transcription, DNA-templated | 103 | 0.00321424260912 |
| 8 | Cellular protein metabolic process | 22 | 0.00640145871122 |
| 9 | Inositol phosphate metabolic process | 6 | 0.0145337518569 |
| 10 | Fc-epsilon receptor signaling pathway | 10 | 0.0145337518569 |
| **miR-423-5p** | | | |
| 1 | Cellular nitrogen compound metabolic process | 521 | 5.17446092617e-43 |
| 2 | Biosynthetic process | 443 | 2.27698527594e-32 |
| 3 | Gene expression | 95 | 3.73939117724e-24 |
| 4 | Cellular protein modification process | 269 | 1.68428072615e-22 |
| 5 | Neurotrophin TRK receptor signaling pathway | 49 | 2.87609306785e-17 |
| 6 | Symbiosis, encompassing mutualism through parasitism | 80 | 3.90332953697e-17 |
| 7 | Viral process | 73 | 5.5484050493e-17 |
| 8 | Cellular protein metabolic process | 69 | 7.40936980223e-15 |
| 9 | Small molecule metabolic process | 233 | 7.11951328031e-12 |
| 10 | Membrane organization | 80 | 2.36236073657e-11 |
| **miR-21-5p** | | | |
| 1 | Cellular nitrogen compound metabolic process | 487 | 4.51382256914e-49 |
| 2 | Biosynthetic process | 403 | 2.64289044294e-33 |
| 3 | Gene expression | 100 | 4.61740114746e-32 |
| 4 | Cellular protein modification process | 265 | 2.12183444247e-30 |
| 5 | Symbiosis, encompassing mutualism through parasitism | 77 | 9.03032199481e-19 |
| 6 | Viral process | 68 | 4.57897937782e-17 |
| 7 | Biological process | 1312 | 9.44848528298e-16 |
| 8 | Macromolecular complex assembly | 105 | 3.83454201018e-13 |
| 9 | Small molecule metabolic process | 214 | 5.13980288765e-13 |
| 10 | Cellular component assembly | 139 | 8.91665824038e-13 |
| **miR-146a-5p** | | | |
| 1 | Cellular nitrogen compound metabolic process | 257 | 4.62040443892e-23 |
| 2 | Biosynthetic process | 207 | 1.31993148889e-13 |
| 3 | Response to stress | 141 | 3.27372920593e-13 |
| 4 | Cellular protein modification process | 134 | 3.08491272962e-12 |
| 5 | Symbiosis, encompassing mutualism through parasitism | 43 | 2.91684650443e-10 |
| 6 | Gene expression | 43 | 1.32826042114e-09 |
| 7 | Viral process | 37 | 8.99775924622e-09 |
| 8 | Immune system process | 98 | 1.73415956135e-08 |
| 9 | Fc-epsilon receptor signaling pathway | 15 | 3.86929755926e-05 |
| 10 | Biological process | 695 | 7.09054084121e-05 |
| **miR-155-5p** | | | |
| 1 | Cellular nitrogen compound metabolic process | 486 | 4.7978838385e-46 |
| 2 | Cellular protein modification process | 263 | 6.00862430213e-28 |
| 3 | Biosynthetic process | 387 | 2.44247779064e-26 |
| 4 | Gene expression | 83 | 2.20176279433e-20 |
| 5 | Small molecule metabolic process | 239 | 2.00129954202e-19 |
| 6 | Catabolic process | 210 | 5.20682183661e-19 |
| 7 | Biological process | 1342 | 2.03605330773e-17 |
| 8 | Cellular protein metabolic process | 63 | 6.30015107107e-14 |
| 9 | Neurotrophin TRK receptor signaling pathway | 41 | 1.93407472582e-13 |
| 10 | Response to stress | 220 | 3.3065880394e-12 |
| **miR-27a-5p** | | | |
| 1 | Cellular nitrogen compound metabolic process | 222 | 5.59789096859e-32 |
| 2 | Biosynthetic process | 174 | 3.35605273872e-18 |
| 3 | Symbiosis, encompassing mutualism through parasitism | 37 | 8.82128992522e-11 |
| 4 | Cellular protein modification process | 103 | 8.82128992522e-11 |
| 5 | Gene expression | 37 | 3.29760686253e-10 |
| 6 | Response to stress | 103 | 7.02007759833e-10 |
| 7 | Viral process | 32 | 2.07741157852e-09 |
| 8 | Cellular component assembly | 67 | 2.41515245029e-09 |
| 9 | Cellular protein metabolic process | 31 | 6.87682420314e-09 |
| 10 | Macromolecular complex assembly | 50 | 1.20217055736e-08 |
| **miR-4433b-5p** | | | |
| 1 | Cellular nitrogen compound metabolic process | 74 | 0.0200409823623 |
| 2 | Post-translational protein modification process | 7 | 0.0496936786784 |
| 3 | Cellular protein modification process | 42 | 0.0496936786784 |
| **miR-4508** | | | |
| 1 | Cellular nitrogen compound metabolic process | 11 | 0.0172822821682 |
| 2 | Negative regulation of metanephric glomerular mesangial cell proliferation | 1 | 0.0305281185125 |
| 3 | Thorax and anterior abdomen determination | 1 | 0.0305281185125 |
| 4 | Positive regulation of metanephric ureteric bud development | 1 | 0.0305281185125 |
| 5 | Depurination | 1 | 0.0305281185125 |
| 6 | Regulation of L-glutamate transport | 1 | 0.0305281185125 |
| 7 | Regulation of organ formation | 1 | 0.0305281185125 |
| 8 | Adrenal cortex formation | 1 | 0.0305281185125 |
| 9 | Posterior mesonephric tubule development | 1 | 0.0305281185125 |
| 10 | DNA metabolic process | 4 | 0.0305281185125 |
| **miR-485-3p** | | | |
| 1 | Cellular nitrogen compound metabolic process | 139 | 1.17107921032e-15 |
| 2 | Blood coagulation | 26 | 2.58420639915e-08 |
| 3 | Platelet activation | 17 | 8.31594310158e-08 |
| 4 | Gene expression | 27 | 8.31594310158e-08 |
| 5 | Biosynthetic process | 99 | 1.37464772865e-05 |
| 6 | Cell adhesion | 41 | 5.77312584094e-05 |
| 7 | Cellular component assembly | 43 | 5.77312584094e-05 |
| 8 | Biological process | 353 | 5.77312584094e-05 |
| 9 | Catabolic process | 56 | 7.6064939504e-05 |
| 10 | Neurotrophin TRK receptor signaling pathway | 13 | 0.000134840260771 |
| **miR-224-5p** | | | |
| 1 | Cellular protein modification process | 215 | 3.03109704614e-28 |
| 2 | Cellular nitrogen compound metabolic process | 340 | 1.08985486169e-26 |
| 3 | Biosynthetic process | 282 | 8.91633397161e-18 |
| 4 | Gene expression | 62 | 5.70556832729e-15 |
| 5 | Response to stress | 166 | 2.23451785876e-09 |
| 6 | Neurotrophin TRK receptor signaling pathway | 30 | 3.66310325551e-09 |
| 7 | Fc-epsilon receptor signaling pathway | 22 | 1.70864937254e-08 |
| 8 | Cellular protein metabolic process | 44 | 1.70864937254e-08 |
| 9 | Cellular component assembly | 101 | 4.56337824756e-08 |
| 10 | Macromolecular complex assembly | 74 | 1.07077583432e-07 |
| **miR-629-5p** | | | |
| 1 | Cellular nitrogen compound metabolic process | 141 | 1.11334282501e-14 |
| 2 | Biosynthetic process | 114 | 5.56282349772e-09 |
| 3 | Gene expression | 25 | 5.31786242726e-06 |
| 4 | Mitotic cell cycle | 19 | 6.19142263792e-05 |
| 5 | Cellular protein modification process | 63 | 0.000553026458121 |
| 6 | DNA metabolic process | 29 | 0.00151203465697 |
| 7 | Cell death | 31 | 0.00425516138739 |
| 8 | Viral process | 17 | 0.00507170146855 |
| 9 | Neurotrophin TRK receptor signaling pathway | 11 | 0.00721267412189 |
| 10 | Symbiosis, encompassing mutualism through parasitism | 18 | 0.0077933116373 |
| **miR-210-3p** | | | |
| 1 | Insulin receptor signaling pathway via phosphatidylinositol 3-kinase | 1 | 0.043075464738 |
| 2 | Positive regulation of steroid hormone biosynthetic process | 1 | 0.043075464738 |
| 3 | Positive regulation of glycogen (starch) synthase activity | 1 | 0.043075464738 |
| 4 | Transcription initiation from RNA polymerase II promoter | 2 | 0.043075464738 |
| **miR-182-5p** | | | |
| 1 | Cellular nitrogen compound metabolic process | 883 | 2.18238724852e-95 |
| 2 | Biosynthetic process | 731 | 3.57511811305e-65 |
| 3 | Cellular protein modification process | 467 | 2.44193555325e-53 |
| 4 | Gene expression | 158 | 6.60653952898e-46 |
| 5 | Symbiosis, encompassing mutualism through parasitism | 142 | 2.80250302877e-38 |
| 6 | Viral process | 127 | 7.10769952814e-36 |
| 7 | Biological process | 2369 | 2.41521753173e-33 |
| 8 | Mitotic cell cycle | 112 | 3.90837003292e-33 |
| 9 | Neurotrophin TRK receptor signaling pathway | 80 | 1.28873072683e-31 |
| 10 | Fc-epsilon receptor signaling pathway | 55 | 1.52197870217e-25 |
